# Quantitative Morphology of Polder Landscape Based on SOM Identification Model: Case Study of Typical Polders in the South of Yangtze River

**DOI:** 10.1155/2022/1362272

**Published:** 2022-05-29

**Authors:** Zhe Li, XinYi Lu, Xiao Han, LiYa Wang, XiaoJian Tang, XiaoShan Lin

**Affiliations:** ^1^School of Architecture, Southeast University, Nanjing, Jiangsu 210096, China; ^2^Architects & Engineers Co., Ltd. of Southeast University, Nanjing, Jiangsu 210096, China

## Abstract

Landscape morphology is a significant area of landscape architecture research. One of the scientific and technological issues in recent landscape morphology research is the use of quantitative analysis technology driven by morphology indexes and computational models to describe, compare, and analyze form features. This article focuses on the form features of the polder landscape, based on existing theoretical and practical achievements in landscape morphology. First, we choose five landscape morphology indexes based on the morphological constituent units of the landscape (elongation, rectangular compactness, concavity, ellipse compactness, and fractal dimension). Then, using the self-organizing map (SOM), we create an identification model for clustering the types of constituent units. The experimental results show that the identification model can classify polder morphology and analyze the distribution of units using typical polders in the Yangtze River's south bank as study cases. This article presents a technical approach to polder landscape morphology classification as well as a reference and developable quantitative analysis method for landscape morphology research.

## 1. Introduction

Landscape morphology is an organic part of the landscape environment that condenses the landscape material space in a plane, which is one of the crucial representatives in distinguishing the landscape feature of different regions [1]. Digital identification and quantitative analysis of representative regional landscapes are of great significance for continuing and reshaping the features of regional landscape morphology [2]. With the support of landscape morphology and typo-morphology theories, developing quantitative analysis technologies suitable for identifying and analyzing polder landscape morphology has become one of the practical problems in the current landscape morphology research.

Contemporary studies on landscape morphology have gradually shown a trend of quantitative research, driven by emerging digital landscape research methods and technologies [3]. The analysis foundation for this type of study is to combine spatial information based on the morphological characteristics of the research object, and then obtain operable and comparable values and intervals. A crucial step is to keep optimizing the morphology index in order to achieve the mathematical translation of objective morphology. Exploring the morphological features of a research question requires building a quantitative analytical model that fits the research question. A landscape morphology analysis model created based on the machine learning model can transform traditional qualitative cognition into quantitative judgment [4]. Related research has been carried out in the fields of landscape classification [5], simulation prediction [6], landscape efficiency, and evaluation [7]. Exploring theoretical methods and technical means in the investigation of a typical zonal landscape morphological feature will effectively respond to the theoretical and practical needs of the refined development of landscape morphology. Meanwhile, it can provide a research basis and technical reference for quantitative research and digital development of landscape morphology [8].

## 2. Relevant Research

### 2.1. Polder Landscape Morphology

**Figure 1:**
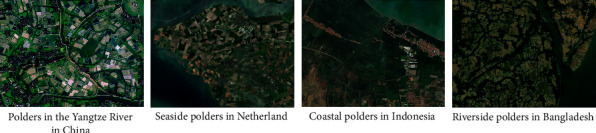
Morphology features of the polders.

Polders are agricultural production environments formed by artificial water conservancy projects, mainly distributed in low-lying and water-rich river banks [9], estuary deltas, and coastal lowlands [10]. In the long-term production evolution process, the morphological features of the wetland polders are integrated with production methods, intertwining the landscape paddy fields, dry land, canals, ponds, and fields and ridges (Figure 1). One of the main difficulties in studying polder landscape is accurately describing and analyzing its morphological features.

In the study of interpretation, historical evolution, planning, and design of polder landscape, the unique landscape morphology of polder has the fundamental significance for regular summary and retrospective tracking. In terms of morphological interpretation and historical evolution, Steenbergen et al. analyzed and illustrated the development and distribution of polder morphology in the Netherlands, and then classified and analyzed the features of the polder from aspects of nature, water conservation, agriculture, and settlement [11]. Missiaen et al. summarized the landscape evolution of polders in northern Belgium [12]. Li et al. used remote sensing images to sort out the spatiotemporal distribution and development of polders in the Dongting Lake Plain in China [13]. In terms of planning and design, Velde and Wit summarized the methods of polder management and planning strategies of polder urban based on morphological research [14]. Nijhuis and Pouderoijen based on the GIS platform to visualize various polder data, introducing a digital orientation to the study and management of the polder landscape morphology [15]. The morphology of the polder also impacts the realization of its production, life, and ecological functions. Optimizing the polder morphology can improve flood management and the capacity for disasters in this area [16, 17]. The landscape morphology element is also a critical research indicator in evaluating the effectiveness of the polder transformation scheme [18] and the value of polder ecosystem services [19].

The polder form has gotten a lot of attention in landscape architecture research because of its distinguishing characteristics and crucial role in polder landscape research. Upgrading morphological classification from empirical cognition to quantitative judgment, as part of the digital landscape research trend, will aid in the promotion of polder landscape research.

### 2.2. Theory and Method of Landscape Morphology

American geographer C. O. Sauer first proposed landscape morphology in 1925 [20]. As an intuitive reflection of landscape environment on a two-dimensional plane, the specific syntagmatic relation and composition methods presented by landscape morphology are an essential carrier for exploring the feature of the landscape. As a branch of morphology research, landscape morphology research closely relates to the trend of morphology research. Since the 1950s, academic circles have paid increasing attention to the inheritance and protection of typical forms. Guided by the theory of morphology and graphical analysis, under the joint influence of Conzen [21] and Muratori-Caniggia [22] produced a morphological research method named typo-morphology, which provides a framework for the study of typical features of complex morphological systems. Specifically, it takes the basic constituent units of the research object's morphology as the entry point and selects geometric morphology indexes to describe the morphological features of the units. Then, identify the research object's morphological types and typical features by analyzing, comparing, and classifying the index value.

Current research continues to verify the applicability of morphology-type analysis to identify morphological features in the built environment and natural environment from multiple perspectives and scales. The perfection and development of digital landscape theory and technical methods also provide new research and practice tools for exploring the feature of morphological [23]. In research of urban morphology [24], settlement pattern [25], and landscape morphology [26] have accumulated a large number of indexes. Meanwhile, statistical and machine learning models are applied to morphology index analysis, expanding the visual analysis capabilities of multidimensional morphological data with large sample sizes. For example, Gil et al. used the K-means algorithm to analyze the typical urban block morphological dataset, realizing the objective classification of block morphology types [27]. SEM [28] and hierarchical clustering [29] also provide practical tools for a comprehensive analysis of morphological data. Machine learning methods such as back propagation neural network [30, 31] and convolutional neural network [32] can also achieve identification and classification of morphological unit types by learning morphological data [33]. On this basis, exploring data-driven landscape morphology research methods with the help of landscape morphology index and mathematical analysis model has positive significance for analyzing the feature of polder morphology.

### 2.3. Application of SOM in Landscape Architecture and Related Research

SOM is an unsupervised machine learning algorithm [34], which does not need to form an evaluation standard by learning samples with existing classification labels. It can perform competitive learning on the potential laws and fundamental attributes of the input samples after independently adjusting the connection weights of the network structure. Therefore, in the analysis without preclassification criteria, often use SOM.

SOM is commonly combined with quantitative indexes and applied in element identification, comprehensive evaluation, and scheme optimization in landscape architecture and related disciplines research. It can be applied to SOM to hierarchical classification studies of building form [35], building space [36], and land use [37] in terms of element recognition. When it comes to comprehensive evaluation, SOM is frequently used to analyze multifactor datasets and solve complex classification problems, such as a comprehensive analysis of tourist motivation and behavior in protected areas [38]. SOM can also be used to investigate relationships between research elements, such as correlations between river ecological indicators [39], pattern associations between different environmental conditions and physiological responses [40], and links between residents' lifestyles and their choice of residence within the city [41]. Using SOM to create a parametric building model can optimize the architectural design scheme [42].

In summary, SOM has substantial autonomy and a low degree of manual intervention. It can adapt to the high-dimensional data [43], which is highly suitable for polder landscape morphological identification's application scenario. In this sense, a polder landscape morphological identification model based on SOM will promote the refinement and intellectual development of polder landscape morphological identification and provide a quantitative analysis method for the research of landscape morphology.

## 3. Methods and Materials

### 3.1. Technology Roadmap

**Figure 2:**
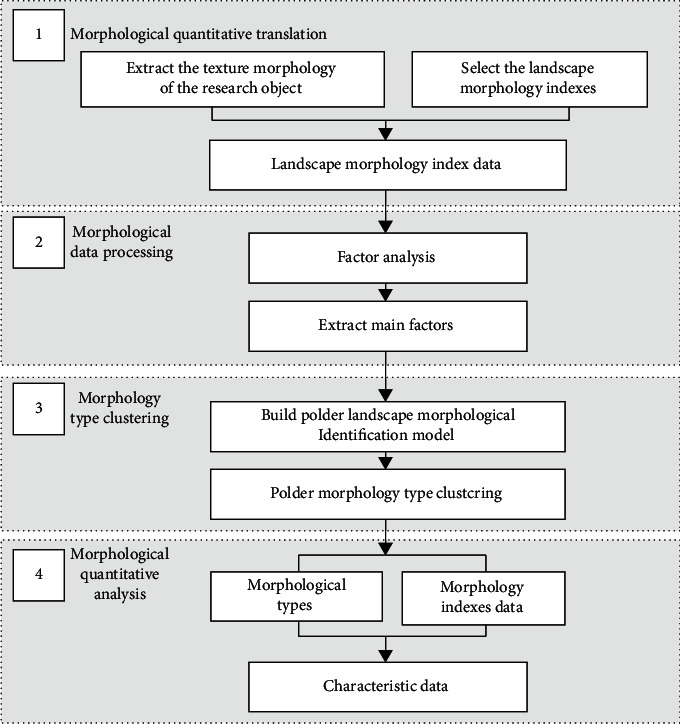
Technology roadmap.

Under the guideline of landscape morphology and typo-morphology theory, we took polder landscape morphology as the research object, attempting to make a quantitative research process to analyze its landscape morphology feature based on the constituent units. The process combined landscape morphology index, SOM, and statistical methods to characterize the overall morphological features and internal differences of the polder units. This article conducted an empirical study on typical polders in the South of the Yangtze River, and the specific research steps are as follows (Figure 2):

1. *Morphological Quantitative Translation*. Select suitable landscape morphology indexes, and convert the polder morphological constituent units into operable, analyzable, and comparable index values.
2. *Morphological Data Processing*. Use factor analysis to integrate the multi-index into several common factors. Then, use common factors value for the subsequent cluster identification of the polder landscape morphology type.
3. *Morphology-Type Clustering*. Use SOM to build a polder landscape morphological identification model. Then, realize the typo-morphology and distribution pattern analysis of the research area through self-learning and clustering of the common factors extracted by factor analysis.
4. *Morphological Quantitative Analysis*. Use the ArcGIS platform to visualize the identification results. Then, establish a polder landscape morphological database, realizing the management, display, retrieval, and quantitative analysis of polder units [44].

### 3.2. Morphology Index Translation

Describing the polder morphology with the landscape morphology index is crucial for identification and classification of the polder morphology types. Based on the two-dimensional plane of the polder landscape constituent morphological unit [45], the morphology indexes selection according to the geometric features of the constituent units. Index selection principles combined scientificity, representativeness, comprehensiveness, and operability. The indexes abstractly translated the two-dimensional morphology of the polder units into the indexes value, providing primary data for subsequent morphology-type classification and feature analysis.

Long-term agricultural production and natural evolution have occurred in the polder landscapes studied in this article. Polder unit boundaries were originally almost regular geometric shapes. The polder units' boundary morphology tends to be tortuous as the water network expands and current scouring occurs, and some water network extends to the interior of the units. The morphology of polder unit boundaries then becomes diversification. A complex and diverse polder landscape morphology is formed by various constituent units. It is difficult to fully reflect the morphological features of the boundary morphology with a single landscape morphology index because the boundary morphology is complex and self-similar. As a result, an index system with comprehensive description dimensions and easy data acquisition is required to describe its morphological features.

We used an interdisciplinary perspective to summarize related index systems in typo-morphology, geoscience, computer vision, and pattern recognition. Then, selected landscape morphology indexes have been empirically effective for two-dimensional morphology measurements in related research. Extended degree, squareness, morphological concavity, and tortuosity were four angles to choose appropriate landscape morphology indexes in this article. Elongation (*λ*) is used to measure extended degree [46], rectangular compactness (S_1_) is used to describe squareness [47], the concavity (*V*) is used to characterize morphological concaveness [48], and the ellipse compactness (*S*_2_) and fractal dimension (*D*) jointly represent tortuosity [49] (Table 1). Then, we obtained the geometric attribute data to calculate the selected indexes value, such as area (*A*), perimeter (*P*), area of minimum bounding rectangle (*A*_min_ref_), area of the minimum convex hull (*A*_min_h_), long axis (*a*_min_ref_), and short axis (*b*_min_ref_). Landscape morphology indexes are linked and complementary to each other, which can jointly describe the boundary morphological features of the polder units.

#### 3.2.1. Elongation (*λ*)

Its geometric meaning is the ratio of the long axis to the short axis of the polder units. In the actual operation, use the ratio of the length of the long side (*a*_min_ref_) to the length of the short side (*b*_min_ref_) of the minimum bounding rectangle to reference it. We use this index to define the extended degree of the shape of the constituent units. The closer the ratio is to 1, the more likely the constituent units appear as equilateral shapes. The ratio is higher, the morphology of the constituent units is more likely to extend in a stripe shape:

(1) $$\begin{array}{c} \lambda =\frac{a_{\text{min\_ref}}}{b_{\text{min\_ref}}}, \end{array}$$

where *a*_min_ref_ represents the length (m) of the long side of the minimum bounding rectangle of the constituent units and *b*_min_ref_ represents the length (m) of its short side.

#### 3.2.2. Rectangular Compactness (*S*_1_)

Rectangular compactness (S_1_) is the ratio of the polder morphological constituent unit area (*A*) to the minimum bounding rectangle area (*A*_min_ref_), and its numerical value ranges from 0 to 1. The closer the ratio is to 1, the more similar the constituent unit boundary to its minimum bounding rectangle. *S*_1_=(*A*/*A*_min_ref_), where *S*_1_ represents the rectangular compactness, *A* represents the area of the polder units (m^2^), and *A*_min_ref_ represents the area of the minimum bounding rectangle of the polder units (m^2^).

#### 3.2.3. Concavity (*V*)

This article use concavity to define the degree of the concave shape of the polder morphological constituent units. A convex hull is the smallest convex polygon that can contain two-dimensional geometry. The concavity value ranges range from 0 to 1. The concavity is more similar to 1, and the boundary of the unit is less concave. When *V* = 0, the shape of the polder unit is a convex polygon:

(2) $$\begin{array}{c} V=\frac{A_{\text{min}\_h}-A}{A_{\text{min}\_h}}, \end{array}$$

where *V* represents the concavity, *A* represents the area of the polder units (m^2^), and *A*_min_h_ represents the minimum convex hull area of the polder units (m^2^).

#### 3.2.4. Ellipse Compactness (*S*_2_)

The ellipse compactness takes the ellipse with the same area (*A*) and the same elongation (*λ*) as the polder shape units as the reference figure. Calculating the ratio of the ellipse perimeter to the perimeter of the constituent units (*P*) can reflect the complexity of the constituent units. Due to the introduction of elongation, this index is more suitable for quantitative analysis of complex boundary patterns. The larger the value of *S*_2_ is, the more tortuous the boundary is:

(3) $$\begin{array}{c} S_{2}=\frac{P}{1.5\lambda +1.5-\sqrt{\lambda }}\sqrt{\frac{\lambda }{A\pi }}, \end{array}$$

where *S*_2_ represents the ellipse compactness, *P* represents the polder unit perimeter (m), *A* represents the polder unit area (m^2^), and *λ* represents the elongation.

#### 3.2.5. Fractal Dimension (*D*)

The shape of the polder units tends to be complex and fragmented in the long-term evolution process, which shows a specific self-similar shape. Thus, we introduce the fractal geometry index to supplement the description of tortuosity. The fractal dimension (*D*) is the most important parameter for describing fractal geometry. Researchers often use it to study irregular shapes in natural and built environments. Since the polder morphology is a two-dimensional figure, the theoretical value of fractal dimension is 1-2. The fractal dimension is closer to 2, the shape of the polder unit is more complex.

(4) $$\begin{array}{c} D=\frac{2\;\ln\left(P\right)}{\ln\left(A\right)}, \end{array}$$

where *D* represents fractal dimension, *P* represents the polder unit perimeter (m), and A represents the polder unit area (m^2^).

### 3.3. Data Processing and Analysis Methods of Landscape Morphology

This article selects factor analysis to integrate the polder landscape morphology index value to avoid repeated information and explore the relationship between different indexes. Factor analysis is a standard multivariate dimensionality reduction statistical method in statistics. Analyzing the internal structural relationship of the original data correlation coefficient matrix converts multiple indicators with complex relationships into a few combinations of random variables, namely, the common factors, which can reproduce the mutual relationship between the original variables and the common factors [50]. The common factor value will be used as input data to identify the morphology type of polder units.

**Figure 3:**
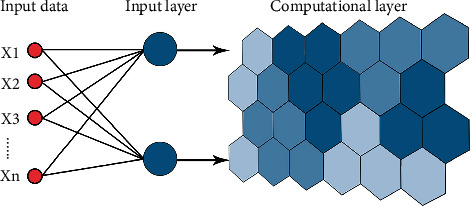
Structure of SOM.

To identify the polder morphological features, selecting an appropriate machine learning model according to the research needs and data characteristics is necessary. We apply unsupervised machine learning for clustering analysis to identify the morphology types of polder units. SOM is a kind of unsupervised artificial neural network similar to the brain neural network. It consists of an input layer and a computational layer. When receiving external information input, different input samples activate various neurons. Through “autonomous learning,” SOM can enable each neuron to form a specific response pattern, thus facilitating the grouping of input samples [51]. Therefore, SOM can directly use the input samples to complete the whole process from network training to sample clustering (Figure 3).

### 3.4. Research Object Extraction

**Figure 4:**
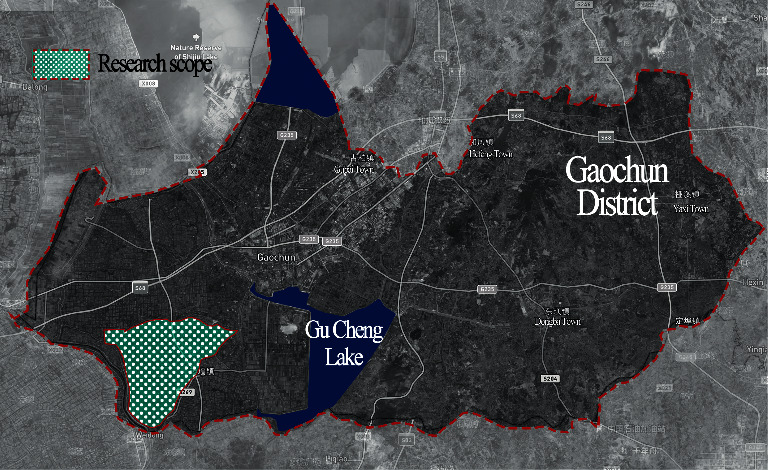
Location and scope of the research object.

We select the typical polders in China as the research object to explore the validity of the polder landscape morphological identification model. The research object is located in Gaochun District, Nanjing city, in the middle and lower reaches of the Yangtze River, with a total area of 3507 hectares (Figure 4). It belongs to the local typical agricultural production environment, with flat terrain and dense water network characteristics. The agricultural production there is closely related to the water network. The polder units formed under the limitation of natural river channels and artificial canals are the primary constituent units of the polder morphology in this area.

**Figure 5:**
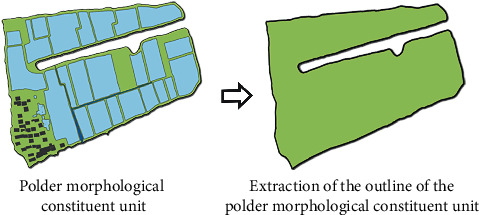
Schematic diagram of morphology extraction and optimization of the research object.

The data extraction for this research is based on the drawings surveyed in 2019. It combined the impact of Landsat 8 satellite remote sensing and field surveys for review and proofreading. We extracted the polder morphological constituent unit boundary as the basis for the study, took the water network and polder dykes as the boundary of the unit morphology, and retained the canals that extend into the polder units (Figure 5).

The study area extracted 381 polder unit samples; the sample size can meet the data requirements of SOM for forming stable and reasonable classification results (≥100). Then, moderately smooth the boundary shape to avoid the measurement error. Store the data on the ArcGIS platform to generate the morphological unit's minimum bounding rectangle and convex hull. After that, extract the fundamental geometric values required for the polder landscape morphology index calculation. The final landscape morphology index statistics of each sample are shown in Table 2. The landscape morphology index statistics of each samples are shown in Table 2.

## 4. Quantitative Results of Polder Landscape Morphology

### 4.1. Data Processing Results

This article used SPSS (26.0) for factor analysis. First, standardize the morphology index data of the polder landscape and conduct moderate analysis by KMO and Bartlett's test. According to the test results, the KMO sampling adequacy is more significant than 0.5, and the significance level of the Bartlett test is less than 0.01, indicating that the tested data are suitable for factor analysis (Table 3).

Extract the first two factors with a characteristic root greater than 1 as the common factors, and the cumulative variance contribution rate is 80.79% (Table 4). It showed that the two common factors contain more than 80% of the original information of the landscape morphology indexes, which is enough to represent the main content of the variable information. The characteristic value of the first factor after rotation is 2.385, and the variance contribution rate is 47.706%. The characteristic value of the second factor after rotation is 1.654, and the variance contribution rate is 33.086%. These two common factors used linear combinations to replace the original landscape morphology indexes. The common factors jointly described the morphology of the polder unit for the following types of clustering (Table 5).

### 4.2. Clustering Analysis Based on SOM

**Figure 6:**
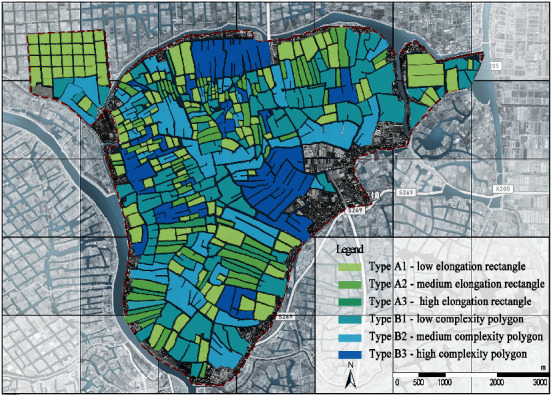
Results of polder landscape morphological clustering.

The SOM model was driven by the two common factors to realize the polder landscape morphology-type clustering. The number of neurons in the input layer, computational layer, and total number of learning times are all important parameters to set when building a model. The input layer's number of neurons is determined by the data it receives. The requirement for morphological clustering is related to the number of neurons in the computational layer. We must determine the specific number combination, universality, and interpretability of the classification results based on the distribution characteristics of the input data. The neural network must reach a point of stability within the allotted learning time. This article used MATLAB R2019b to create a SOM model to achieve the clustering of the polder morphology types after several experiments to determine the parameters. Connect the results to the ArcGIS platform for visual display, and create a polder landscape morphological database to serve as the data and image foundation for polder morphology-type analysis. The specific clustering results are shown in Figure 6.

### 4.3. Morphology Clustering Results of Polder Landscape

From the SOM cluster results, the polder units' morphological features in the same clusters have consistency, and the features in different clusters have significant differences. A comprehensive analysis of the identification results of polder landscape morphology shows that the average value of the landscape morphology index of each category has a logical law, which indicates that SOM can autonomously make the clustering results of polder morphology types highly interpretable (Table 6).

1. From an individual perspective, rectangular and polygonal units constitute the polder feature. From the landscape morphology index perspective, the morphological constituent units identified by SOM can classify into two categories: rectangular (Type A) and polygonal (Type B). The former has high squareness, low concavity, and low tortuosity, so this type of unit presents a relatively simple and square overall shape. The latter has low squareness, high concavity, and high tortuosity, so the morphological features are complex and tortuous. In the rectangular unit, due to the difference in elongation, the morphology type of the constituent units can further divide into low elongation rectangle (Type A1), medium elongation rectangle (Type A2), and high elongation rectangle (Type A3). In polygonal types, the concavity, ellipse compactness, and fractal dimension increase simultaneously as the rectangular compactness decreases. The morphology type of constituent units can divide into low complexity polygon (Type B1), medium complexity polygon (Type B2), and high complexity polygon (Type B3). In terms of the morphology of polder units, their boundary shapes tend to be complex, and the number and length of concave channels gradually increase.
2. From an overall perspective, the distribution pattern of the polder units is closely linked to the morphological feature. Regarding the proportion and distribution of morphological units, the number of rectangular and polygonal units is basically the same: rectangular units accounted for 51.4% of the total polder morphological units, and polygonal units accounted for 48.6%. From the perspective of morphological distribution, the morphological composition of the polder shows the combination of different types of morphological units, which makes the polder in the studied area present a multicomplex morphological feature.

## 5. Analysis and Discussion

In this article, the identification of the polder landscape morphology based on the SOM identification model can effectively classify the types of polder landscape morphology in the research area. The results have application value for quantitative research and analysis on the morphology of the polder landscape. This technical route is suitable for in-depth development and horizontal expansion in the field of landscape morphology research.

1. At the level of research effectiveness, the SOM identification model effectively classified the polder morphology in the studied area and obtained relatively stable six types of polder units, realizing the division of polder landscape morphology types in the individual perspective. We integrated and applied five landscape morphology indexes such as elongation and rectangular compactness, which can effectively translate the polder landscape morphology of the studied area. Thus, this technical system has specific reference significance and application value for the quantitative analysis of complex and wide-area landscape morphology such as polder.
2. At the application level, we quantitatively translated and clustered the polder unites to achieve advanced research on polder landscape morphology. From an individual perspective, the technical system can quantitatively study the geometric properties of various polder units and the threshold of the morphology index. From an overall perspective, the technical system can present the units' composition features of the polder landscape morphology. Combined with morphology index value in different periods, it can play a role in tracking the morphological evolution of polders.
3. At the level of research expansion, the technical route can further expand research and perfect. The landscape morphology indexes used in this article mainly focus on the two-dimensional plane. Follow-up research can further be developed by selecting landscape morphology indexes from other perspectives such as facade and space. It can also expand and enrich the research samples to improve the cognition of the polder landscape morphology type at different research levels and scales.

## 6. Conclusions and Prospects

This article took the quantitative analysis method of polder landscape morphology as the critical issue, constructed an index system to describe its morphological features, and formed an identification model based on SOM to classify its types. Taking the typical polders in the South of Yangtze River as the research object, we explored and verified machine learning classification, visualization, and quantitative analysis methods of the morphological features of the polder landscape. This article realized the research and development of automatic identification and quantitative judgment of polder landscape morphology, providing an analysis method and analysis technology that can be referred to and developed for landscape architecture morphology research.

Combining quantitative indexes and machine learning models is one of the quantitative research methods of landscape architecture. For landscape morphology research, the quantitative research process can not only enrich the theory and technical system, but also provide a data basis for analysis, comparison, summary, and traceability. Computer technology has brought a series of cutting-edge analysis methods such as machine learning to the development of landscape architecture. While promoting in-depth scientific research, the technology promotes the iterative development of professional theories, methods, and technologies. Thus, it is an inevitable choice for landscape architecture's informatization and intellectual development.

## Figures and Tables

**Table 1:** Summary table of morphology indexes and schematic diagram.

Name of morphology index	Elongation	Rectangular compactness	Concavity	Ellipse compactness	Fractal dimension
Formula of morphology indexes	*λ*=*a*_min_ref_/*b*_min_ref_	*S*_1_=*A*/*A*_min_ref_	*V*=((*A*_min_*h*_ − *A*)/*A*_min_*h*_)	$S_{2}=\left(P/\left(1.5\lambda +1.5-\sqrt{\lambda }\right)\right)\sqrt{\lambda /A\pi }$	*D*=2ln(*P*)/ln(*A*)
Schematic diagram of morphology indexes	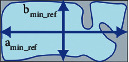	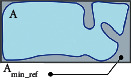	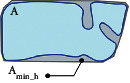	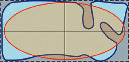	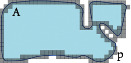

**Table 2:** Descriptive statistics of landscape morphology indexes.

Landscape morphology index	Maximum	Minimum	Average value	Standard deviation
Elongation	11.10	1.00	2.42	1.65
Rectangular compactness	0.99	0.42	0.78	0.12
Concavity	0.67	0.00	0.10	0.12
Ellipse compactness	4.28	1.00	1.22	0.30
Fractal dimension	1.47	1.24	1.30	0.04

Note. Sample size *n* = 381.

**Table 3:** KMO and Bartlett's test.

KMO sampling adequacy		0.580

Bartlett's test	Approximate chi-square	894.835
	df	10
	Sig.	.000

**Table 4:** Total variance explained.

Initial eigenvalues				Extraction sums of squared loading			Rotation sums of squared loading		
Component	Total	% of variance	Accumulative %	Total	% of variance	Accumulative %	Total	% of variance	Accumulative %
1	2.520	50.397	50.397	2.520	50.397	50.397	2.385	47.706	47.706
2	1.520	30.396	80.792	1.520	30.396	80.792	1.654	33.086	80.792
3	.525	10.504	91.296						
4	.247	4.949	96.245						
5	.188	3.755	100.000						

**Table 5:** Common factor coefficient matrix.

	Landscape morphology index	Before rotation	After rotation
Factor 1	Zscore (Rectangular compactness)	−0.845	−0.860
	Zscore (Concavity)	0.752	0.845
	Zscore (Fractal dimension)	0.905	0.901
Factor 2	Zscore(Elongation)	0.920	0.936
	Zscore (Ellipse compactness)	0.671	0.848

**Table 6:** The results of polder landscape morphology types and the average value of morphology indexes.

Type A—rectangular units	Type A1—low elongation rectangle	*n* = 119	Type A2—medium elongation rectangle	*n* = 61	Type A3—high elongation rectangle	*n* = 16
	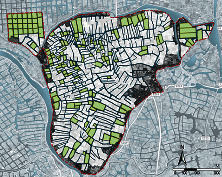	*λ* = 1.68	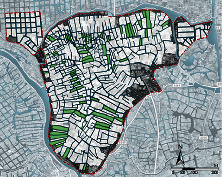	*λ* = 3.51	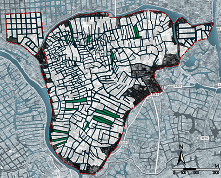	*λ* = 7.83
		*S*_1_ = 0.87		*S*_1_ = 0.81		*S*_1_ = 0.80
		*V* = 0.03		*V* = 0.07		*V* = 0.12
		*S*_2_ = 1.09		*S*_2_ = 1.08		*S*_2_ = 1.04
		*D* = 1.28		*D* = 1.28		*D* = 1.37
Type B— polygonal units	Type B1—low complexity polygon	*n* = 123	Type B2—medium complexity polygon	*n* = 41	Type B3—high complexity polygon	*n* = 21
	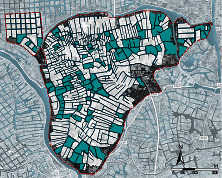	*λ* = 1.59	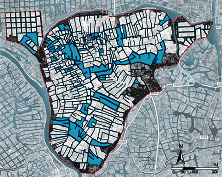	*λ* = 2.48	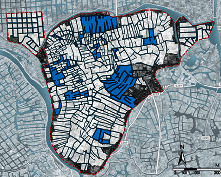	*λ* = 2.20
		*S*_1_ = 0.72		*S*_1_ = 0.65		*S*_1_ = 0.51
		*V* = 0.10		*V* = 0.21		*V* = 0.47
		*S*_2_ = 1.25		*S*_2_ = 1.49		*S*_2_ = 1.95
		*D* = 1.28		*D* = 1.33		*D* = 1.35

## Data Availability

The data presented in this study are available on request from the corresponding author. The data are proprietary or confidential in nature and may only be provided with restrictions.
